# Identification of long non-coding RNA and circular RNA associated networks in cellular stress responses

**DOI:** 10.3389/fgene.2023.1097571

**Published:** 2023-02-10

**Authors:** Xiuzhi Li, Jingxin Li, Ge Shan, Xiaolin Wang

**Affiliations:** ^1^ Department of Clinical Laboratory, The First Affiliated Hospital of USTC, School of Basic Medical Sciences, Division of Life Science and Medicine, University of Science and Technology of China (UTSC), Hefei, Anhui, China; ^2^ Department of Pulmonary and Critical Care Medicine, Regional Medical Center for National Institute of Respiratory Diseases, Sir Run Run Shaw Hospital, School of Medicine, Zhejiang University, Hangzhou, China

**Keywords:** er stress, glucose deprivation, lncRNA, circRNA, miRNA, ceRNA, RBP, network

## Abstract

Mammalian cells employ various adaptive responses to cope with multiple stresses to maintain homeostasis. Functional roles of non-coding RNAs (ncRNAs) in response to cellular stresses have been proposed, and systematical investigations about the crosstalk among distinct types of RNAs are required. Here, we challenged HeLa cells with thapsigargin (TG) and glucose deprivation (GD) treatments to induce endoplasmic reticulum (ER) and metabolic stresses, respectively. Ribosomal RNA (rRNA)-depleted RNA sequencing (RNA-seq) was then performed. Characterization of the RNA-seq data revealed a series of differentially expressed long non-coding RNAs (lncRNAs) and circular RNAs (circRNAs) with parallel changes responsive to both stimuli. We further constructed the lncRNA/circRNA-mRNA co-expressing network, competing endogenous RNA (ceRNA) network in the lncRNA/circRNA-miRNA-mRNA axis, and lncRNA/circRNA-RNA binding protein (RBP) interactome map. These networks indicated the potential *cis* and/or *trans* regulatory roles of lncRNAs and circRNAs. Moreover, Gene Ontology analysis demonstrated that these identified ncRNAs were associated with several essential biological processes known to be related to cellular stress responses. In conclusion, we systematically established functional regulatory networks of lncRNA/circRNA-mRNA, lncRNA/circRNA-miRNA-mRNA and lncRNA/circRNA-RBP to perceive the potential interactions and biological processes during cellular stresses. These results provided insights in ncRNA regulatory networks of stress responses and the basis for further identification of pivotal factors involved in cellular stress responses.

## Introduction

Eukaryotic cells possess an extraordinary capacity to adapt to extra- or intracellular stimuli such as endoplasmic reticulum (ER) stress, heat shock, UV light, oxidative stress and nutrient starvation (Pakos-Zebrucka et al., 2016; Galluzzi et al., 2018; Bhardwaj et al., 2020). To cope with multiple stimuli, cells activate distinct cellular responses including the regulation of gene transcription, the DNA damage response (DDR), the unfolded protein response (UPR), mitochondria stress signaling and autophagy (Eisner et al., 2018; Galluzzi et al., 2018; Vihervaara et al., 2018; Hetz et al., 2020; Larsen et al., 2022). Constant exposures to stresses contribute to various diseases, such as diabetes mellitus, neurodegeneration, cardiovascular disorders and cancers (Urra et al., 2016; Ren et al., 2021; Larsen et al., 2022). Although a range of factors play potent roles in cellular stress responses, and their interactions have been established, the construction of distinct RNA regulatory networks is still in demand (Ermolaeva and Schumacher, 2014; Frakes and Dillin, 2017; Galluzzi et al., 2018; Melber and Haynes, 2018).

ER stress is characterized by the accumulation of unfolded or misfolded proteins in the ER lumen, which triggers the UPR to restore protein homeostasis (Urra et al., 2016; Hetz et al., 2020). Several chemicals including thapsigargin (TG), tunicamycin and dithiothreitol, could induce ER stress and activate the UPR (Feng et al., 2014; Keestra-Gounder et al., 2016). Under ER stress, mammalian cells make efforts in cellular responses *via* three distinct stress sensors: IRE1α, ATF6, and PERK (Hetz et al., 2020). For example, the activated IRE1α selectively splices the *XBP1* mRNA to generate *XBP1s*, and XBP1s protein could regulate the transcription of target genes that are involved in protein folding (Yoshida et al., 2001; Hetz et al., 2020). Glucose deprivation (GD) is another stress that is accompanied by the metabolic oxidative stress, as glucose is the major energy source that generates ATP *via* glycolysis or mitochondrial oxidative metabolism (Zhang C. S. et al., 2017; Ren and Shen, 2019). Upon GD treatment, some primary metabolic pathways are quickly triggered to recover energy homeostasis and promote cell survival (Le et al., 2012; Li et al., 2022). For example, AMP-activated protein kinase (AMPK) mediated catabolism is activated, and the mammalian target of rapamycin (mTOR) mediated anabolism is decreased in response to GD-induced metabolic changes (Zhang C. S. et al., 2017; González et al., 2020).

In mammals, the majority of the genome is transcribed into a variety of non-coding RNAs (ncRNAs) with regulatory roles in physiology and diseases (Hu and Shan, 2016; Quinn and Chang, 2016; Wang et al., 2022). Lines of evidence have demonstrated that ncRNAs are indispensable regulators in various biological processes including transcriptional regulation, modulating alternative splicing, chromatin remodeling, and protein transportation (Quinn and Chang, 2016; Wang et al., 2022). MicroRNAs (miRNAs) are small ncRNAs that function mainly by binding to the 3′ untranslated regions (3′ UTRs) of targets to repress translation (Bartel, 2004; Jonas and Izaurralde, 2015). For example, *miR-3648* is induced under ER stress and decreases the APC2 level to promote cell proliferation (Rashid et al., 2017). Additionally, several miRNAs could exert their functions by targeting the 5’ UTRs or coding regions of the corresponding genes (Liang et al., 2014; Li et al., 2016; Wang et al., 2022). Long ncRNAs (lncRNAs) are endogenously expressed RNA transcripts longer than 200 nucleotides (Quinn and Chang, 2016; Sun et al., 2018). LncRNAs have a broad range of biological functions, such as modulating alternative splicing and regulating translation (Quinn and Chang, 2016; Sun et al., 2018; Wang et al., 2022). The lncRNA *LASTR* is upregulated in hypoxic breast cancer and increases the fitness of breast cancer cells by regulating the activity of the U4/U6 recycling factor SART3 (De Troyer et al., 2020). The lncRNAs such as *5S-OT* and *MALAT1* modulate alternative splicing (Hu et al., 2016; Zhang X. et al., 2017). The lncRNA *Caren* represses the translation of Hint1 to inactivate DDR and activate mitochondrial biogenesis to antagonize heart failure (Sato et al., 2021). Circular RNAs (circRNAs) are covalently closed RNA molecules that are generated by back-splicing or other RNA circularization mechanisms (Kristensen et al., 2019; Liu et al., 2020; Chen et al., 2022). Most circRNAs are thought to be non-coding and exert their functions by mechanisms such as acting as miRNA sponges, modulating RNA binding proteins (RBPs), and regulating gene transcription (Hansen et al., 2013; Memczak et al., 2013; Li et al., 2015; Yang et al., 2017; Kristensen et al., 2019; Gao et al., 2020; Liu et al., 2020; Wang et al., 2021; Chen et al., 2022). The circRNA *cPWWP2A* retards diabetes-induced microvascular dysfunction by sequestering *miR-579* from its targets, angiopoietin 1, occludin, and SIRT1 (Liu et al., 2019). *CircACC1* is upregulated during metabolic stress and enhances the enzymatic activity of AMPK to modulate both glycolysis and fatty acid β-oxidation (Li et al., 2019). Accumulating evidence has demonstrated that ncRNAs could play diverse roles through forming complex regulatory networks including feedback loops, ceRNA networks, co-expressed networks, and RNA-protein complexes (Fu, 2014; Anastasiadou et al., 2018). For example, Kleaveland and colleagues characterized a ceRNA network centered on four ncRNAs—one lncRNA (*Cyrano*), one circRNA (*CDR1as*), and two miRNAs (*miR-7* and *miR-671*) by using a panel of mouse knockouts (Kleaveland et al., 2018). Additionally, *miR-143* and *miR-145* are co-expressed miRNAs that have been extensively studied as potential tumor suppressors (Kent et al., 2014). The well-characterized lncRNA *NEAT1*, binds to various proteins such as TDP-43, KCNAB2, and WDR5, to exert its functional roles (Ahmed et al., 2018; An et al., 2018). Although several classes of ncRNAs including miRNAs, lncRNAs and circRNAs have been reported to play vital roles in response to cellular stresses (Leung and Sharp, 2010; Quinn and Chang, 2016; Chen et al., 2022), identifying more functional ncRNAs and constructing the interacted networks would provide further insights into stress responses.

Numerous studies have focused on one particular cellular stress (Galluzzi et al., 2018; González-Quiroz et al., 2020; Larsen et al., 2022), and to investigate functional ncRNAs in response to more than one stress condition, we performed high-throughput RNA sequencing (RNA-seq) of HeLa cells under TG or GD treatment, and identified differentially expressed lncRNAs and circRNAs in response to stresses. Then, we further established lncRNAs and circRNAs associated networks to characterize key regulators and provide novel insights into cellular stress responses.

## Materials and methods

### Cell culture

HeLa cells were purchased from the American Type Culture Collection (ATCC, http://www.atcc.org) and authenticated by short-tandem-repeat (STR) profiling. They were cultured under standard conditions with DMEM (Gibco, 11995065) containing 10% FBS (CLARK, FB25015), and 1% penicillin/streptomycin (Beyotime, C0222) at 37°C with 5% CO_2_. HeLa cells were determined with a PCR-based method and DAPI staining to ensure no contamination of *mycoplasma*.

### Cellular stress treatments

To induce ER stress, HeLa cells were cultured with the DMEM medium containing 300 nM TG (Sigma, T9033) for 6 h. For glucose deprivation, cells were cultured with DMEM without glucose (Gibco, 11966025) at 37°C for 6 h.

### Library preparation for ribo-minus RNA-seq

Total RNA was extracted by TRIzol reagent (Invitrogen, 15596026) according to the manufacturer’s instructions. The concentration and quality of extracted RNAs were verified by Nanodrop and gel electrophoresis, respectively. Libraries were constructed by the TruSeq Ribo Profile Library Prep Kit (Illumina, RPHMR12126) according to the manufacturer’s instructions. In brief, 10 μg total RNA was depleted rRNA with the Illumina Rio-Zero Gold Kit (Illumina, MRZE724) and next purified for end repair and 5′ adaptor ligation. Then, the reverse transcription was performed with random primers containing the 3′ adaptor and randomized hexamer sequences. Finally, complementary DNA (cDNA) was purified and amplified with a Thermal Cycler. The PCR products of 300–500 base pairs (bp) were purified, quantified and stored at −80°C before sequencing. The libraries were subjected to 150-nt paired-end sequencing generating a depth of 50–100 million read pairs with an Illumina Novaseq platform (Novogene).

### Transcriptome data analysis

For data processing, the adaptors were trimmed with Cutadapt to obtain clean reads. The data quality was then checked with FastQC and the low-quality (Q value ≤ 20) reads were removed. The remaining reads were subsequently aligned to the human reference genome (hg19) with Bowtie2 (-v 1). For linear RNAs including lncRNAs and mRNAs, the corresponding reads were counted with BEDtools and read per million (RPM) was used to calculate levels for lncRNAs and mRNAs. LncRNAs with an average RPM ≥0.1 were used for further analysis and the DE lncRNAs were determined by DEseq2 with a criterion of fold change ≥2 or ≤0.5 and *p*-value < 0.05. The DE mRNAs were determined by DEseq2 with the cutoff (the average RPM ≥10, fold change ≥2 or ≤0.5 and *p*-value < 0.05). For circRNA prediction, find_circ and CIRI2 were applied to identify high-confidence BSJs with default parameters. Only circRNA candidates predicted by both pipelines were used for further investigations and CIRI2-annotated BSJ reads (BSJ reads ≥2) were used to calculate circRNA levels. The DE circRNAs were determined by DEseq2 with the cutoff (fold change ≥2 or ≤0.5, and *p*-value < 0.05).

### PCR reactions

cDNA was synthesized from 500 ng total RNA with the GoScript Reverse Transcription System (Promega, A5000) according to the manufacturer’s protocol. For RT-PCR gels of *XBP1* and *FST* mRNAs, amplification was performed with 30 cycles. Real-time quantitative PCR (RT-qPCR) was carried out with GoTaq SYBR Green qPCR Master Mix (Promega, A6001) on a QuantStudio Applied Biosystems (Thermo) according to standard procedures. All amplification curves reached the stationary stage before 35 cycles and the readings of the Ct value were obtained at the exponential stage. *ACTB* mRNA was used as an internal control. All PCR products were sequenced for confirmation and all primer sequences were included in Supplementary Table S3.

### Plasmid construction and cell transfection

All plasmids for the luciferase reporter system were constructed with recombinant methods (Vazyme, c113-02). *PRDM1* 3′ UTR containing the binding sites of *miR-9-5p* and *SOX12* 3′ UTR containing the *miR-744-5p* binding sequences were PCR-amplified from the cDNA of HeLa cells and then inserted into the Firefly luciferase reporter vector pGL3-control (Promega, E1741) between XbaI (Thermo, FD0685) and FseI (NEB, R0588V) double-digested sites. Small interfering RNAs (siRNAs) targeting *lncSLC25A1*, *TINCR* and *circBANP* BSJ were synthesized by GenePharma (Shanghai, China). Transfection of plasmids and siRNAs was performed with Lipofectamine 2000 (Invitrogen, 11668019) according to the manufacturer’s protocol. Oligonucleotide sequences for primers used in plasmid construction and siRNAs are included in Supplementary Table S3.

### Construction of the co-expression network

The lncRNA/circRNA-mRNA co-expression network was constructed according to the expression levels in our dataset. Briefly, 51 lncRNAs, 39 circRNAs, and 279 mRNAs sensitive to TG and GD treatments were used to construct the network with a criterion of Spearman R ≥ 0.95 or ≤ -0.95 and *p*-value < 0.01. The constructed network consisted of 29 lncRNAs, 20 circRNAs, and 131 mRNAs, and was visualized with Cytoscape (https://cytoscape.org/).

### Construction of the ceRNA network

The ceRNA network was constructed based on the lncRNA/circRNA-miRNA-mRNA axis. The lncRNA-miRNA, circRNA-miRNA, and mRNA-miRNA interactions were predicted with TargetScanHuman (https://www.targetscan.org/vert_72/). Briefly, mRNAs, lncRNAs, or circRNAs with at least two putative binding sites for the individual miRNA were used to construct the network.

### Dual-luciferase reporter assay

Briefly, 1×10^6^ HeLa cells were co-transfected with 30 pmol siRNAs, 1 μg pGL3 Firefly luciferase plasmids and 100 ng pRL Renilla luciferase reporter vector (Promega, E2261). After transfection for 48 h, cells were lysed with passive lysis buffer on ice for 20 min and the luciferase activity was performed with Dual Luciferase Reporter Assay System Kit (Promega, E1910) according to the manufacturer’s protocol. The Firefly luciferase activities were measured and normalized to Renilla luciferase activities (F/R).

### The RNA-RBP network

The RNA-RBP network was constructed based on the lncRNA-RBP and circRNA-RBP interactions predicted by RBPmap (http://rbpmap.technion.ac.il/). The lncRNA/circRNA-RBP interaction was determined by a criterion of the individual RNA sequence containing more than 2 motifs for RBP of interest (*p*-value < 10E-4). The RNA-RBP interactome map consisted of 46 lncRNAs, 17 circRNAs and 77 RBPs, and was visualized with Cytoscape.

### Fluorescence *in situ* hybridization (FISH)

FISH was carried out as previously described with minor modifications (Hu et al., 2016). RNA probe antisense to *linc00612* was generated by the Transcript Aid T7 High Yield Transcription Kit (Thermo, K0441) with the corresponding insertion into the T vector (Promega, A3600) as a template. The probe was then labeled with Alexa Fluor546, by using the ULYSIS Nucleic Acid Labeling Kit (Thermo, U21652), which added a fluor on every G of the probe to amplify the fluorescence intensity. The primers for the antisense probe amplification were included in Supplementary Table S3. HeLa cells were fixed with 4% PFA for 10 min at room temperature after washing with PBS twice. RNA probes were denatured at 80°C for 10 min and placed on ice immediately. Fixed cells were incubated with RNA probes mixed with 20 ng/μL human Cot-1 DNA (Invitrogen, 15279011), 500 ng/μL yeast total RNA (Invitrogen, AM7118) and 10 units/mL RNase inhibitor (Promega, N2615) in 2 × hybridization buffer (4 × SSC, 40% dextran sulfate) at 37°C for 15–17 h, protected from light. After two 10-min washes in SSCT (2 × SSC and 0.4% Tween 20) buffer, nuclei were stained with DAPI (Sigma, F6057). Finally, images were captured using the LSM 980 confocal microscope (Zeiss).

### eCLIP-seq data analysis

Published eCLIP-seq data were obtained from Gene Expression Omnibus (GEO) database under the following accession numbers: GSE91952 (EIF4G2), GSE126263 (MSI1), GSE71096 (SRSF10), GSE69153 (RC3H1) and GSE107768 (FUBP3, DAZAP1, HNRNPA0 and PABPC4).

### GO analysis

GO analysis of the DE mRNAs was performed using the GOrilla web-server with default parameters (http://cbl-gorilla.cs.technion.ac.il) (Eden et al., 2009). For data visualization, the plots were generated by the ggplot2 package in R software.

### Statistical analysis

The physiological experiments were carried out in triplicates (N = 3), and statistical analysis of the data was performed with the two-tailed Student’s t-tests. Data were present as the mean from three independent experiments with SEM. The RNA-seq was performed with four replicates and statistical analysis for analysis was calculated by DEseq2.

## Results

### Global transcriptome analysis for cells under TG and GD treatments

To identify stress-related ncRNAs, we performed ribosomal RNA (rRNA) depleted RNA sequencing (RNA-seq) of human HeLa cells treated with TG (an ER stress inducer) or GD (a metabolic oxidative stress inducer). XBP1s is a well-characterized marker for ER stress and Follistatin (FST) is upregulated in response to GD (Yoshida et al., 2001; Gao et al., 2014). We first examined the levels of *XBP1s* and *FST* mRNAs, and found that *XBP1s* and *FST* detected by RT-PCR with specific primers were significantly increased upon TG and GD treatment, respectively (Supplementary Figure S1A). Then, we performed the bioinformatics to analyze lncRNAs and circRNAs in our dataset (Supplementary Figure S1B). To identify high-confidence back-splicing junctions (BSJs) of circRNAs, two published pipelines including CIRI2 (Gao et al., 2018) and find_circ (Memczak et al., 2013) were applied to annotate circRNAs (Supplementary Figure S1B). CircRNA candidates overlapped in these two pipelines were used for further investigations. Then, the differentially expressed (DE) circRNAs and lncRNAs were analyzed (Supplementary Figure S1B). Principal component analysis (PCA) plots with all identified transcripts also revealed that four biological replicates clustered together, and the controls, TG and GD groups were clearly separated (Supplementary Figure S1C), indicating reliability of RNA-seq and data analyses.

### Differentially expressed lncRNAs and circRNAs in stress responses

**FIGURE 1:**
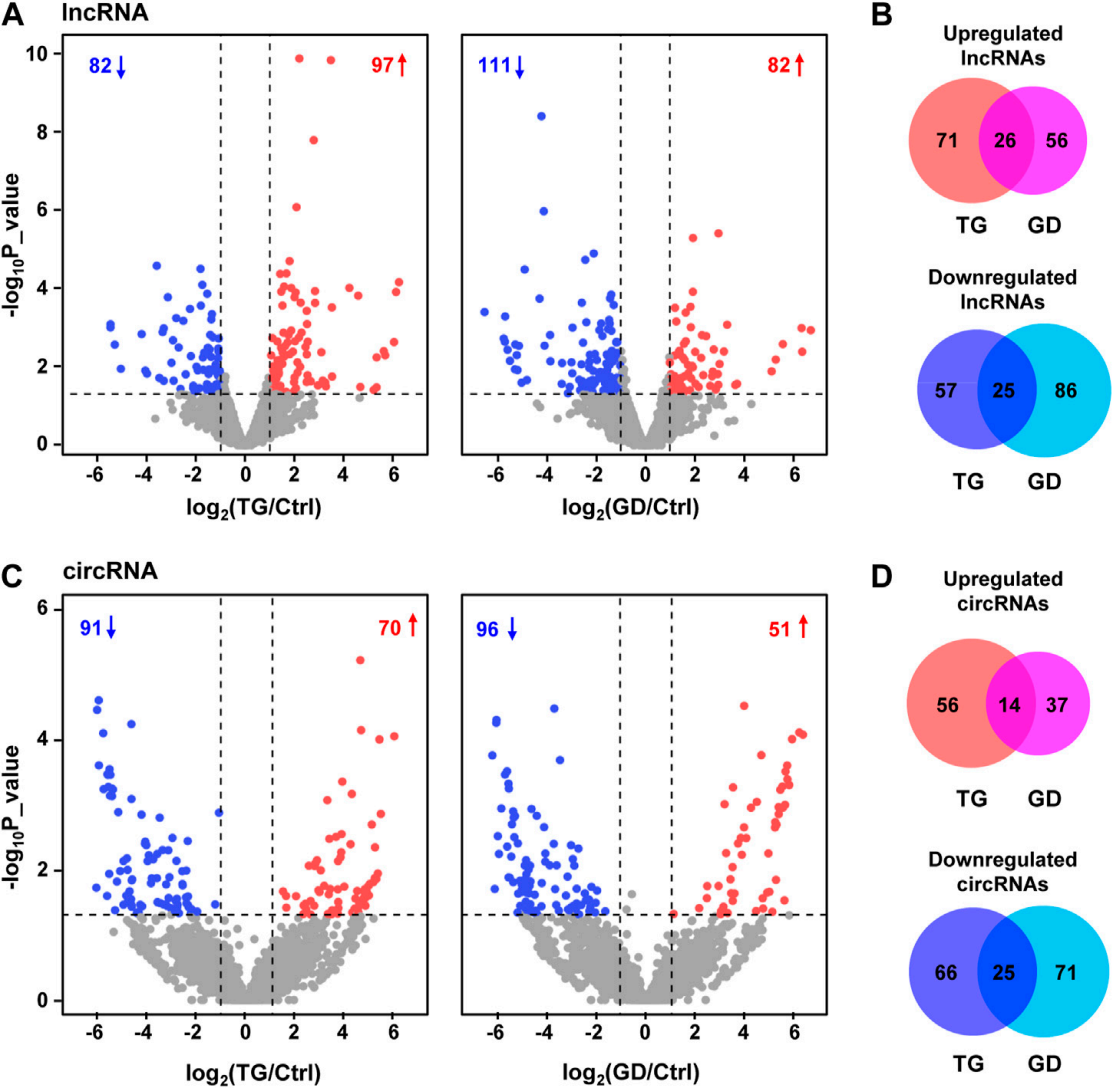
Transcriptome analyses of HeLa cells treated with TG and GD **(A)** Volcano plots displaying the differentially expressed lncRNAs in HeLa cells treated with TG and GD. Blue dots represent significantly downregulated lncRNAs and red dots represent significantly upregulated lncRNAs. Gray dots represent unchanged lncRNAs **(B)** Venn diagram revealing the overlap of dysregulated lncRNAs under TG and GD treatments **(C)** Volcano plots displaying the differentially expressed circRNAs in HeLa cells treated with TG and GD. Blue dots represent significantly downregulated circRNAs and red dots represent significantly upregulated circRNAs. Gray dots represent unchanged circRNAs **(D)** Venn diagram revealing the overlap of dysregulated circRNAs under TG and GD treatments.

**FIGURE 2:**
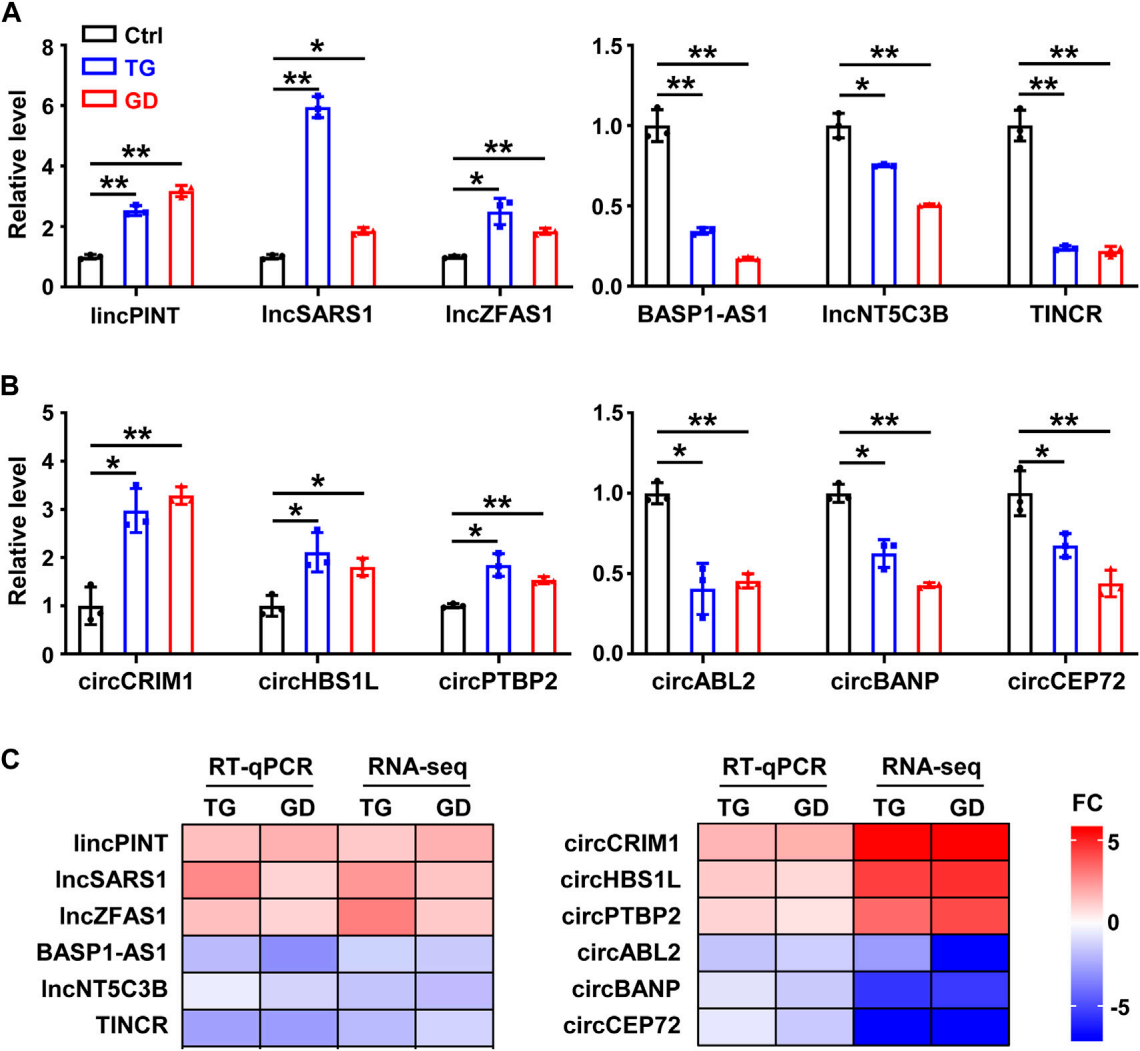
Experimental validation of lncRNAs and circRNAs in HeLa cells **(A)** RT-qPCR analysis of six differentially expressed lncRNAs in HeLa cells treated with TG and GD **(B)** RT-qPCR analysis of six differentially expressed circRNAs in HeLa cells treated with TG and GD **(C)** Heatmaps for the comparison of the RT-qPCR and RNA-seq. Red boxes represent upregulated candidates after TG or GD treatment and blue boxes represent downregulated candidates. FC, fold change. Data are representative of three independent experiments and shown as mean ± SEM. *p < 0.05; **p < 0.01 by two-tailed Student’s t-test.

We characterized 2,406 lncRNAs in our dataset, among which 179 (97 upregulated, 82 downregulated) and 193 (82 upregulated, 111 downregulated) DE lncRNAs were identified (fold change ≥2 or ≤0.5, *p*-value < 0.05) upon TG and GD treatments, respectively (Figure 1A and Supplementary Table S1). Among them, 26 and 25 lncRNAs were significantly increased and decreased in response to both TG and GD treatments (Figure 1B). Simultaneously, we discovered ∼1‰ BSJ reads in our dataset and identified a total of 6531 circRNAs (BSJ reads ≥2) (Supplementary Figure S1D). We determined 161 (70 upregulated, 91 downregulated) and 147 (51 upregulated, 96 downregulated) DE circRNAs (fold change ≥2 or ≤0.5, *p*-value < 0.05) upon TG and GD treatments, respectively (Figure 1C and Supplementary Table S2). Among them, 14 and 25 circRNAs were markedly increased and decreased in response to both TG and GD treatments (Figure 1D). Then, we focused on 51 lncRNAs and 39 circRNAs both dysregulated in response to ER and metabolic stresses (Figures 1B, D). We randomly selected 6 lncRNAs and 6 circRNAs among them for experimental validation. Real-time quantitative PCR (RT-qPCR) analysis demonstrated that *lincPINT*, *lncSARS1*, *lncZFAS1*, *circCRIM1*, *circHBS1L*, and *circPTBP2* were significantly increased after TG and GD treatments (Figures 2A, B). Conversely, *BASP1-AS1*, *lncNT5C3B*, *TINCR*, *circABL2*, *circBANP*, and *circCEP72* were significantly decreased upon TG and GD treatments (Figures 2A, B). For these candidates examined, RT-qPCR-mediated verification was highly consistent with the RNA-seq data (Figure 2C). Taken together, the DE lncRNAs and circRNAs might play essential roles in coping with cellular stresses.

### Co-expression network for lncRNA/circRNA/mRNA

**FIGURE 3:**
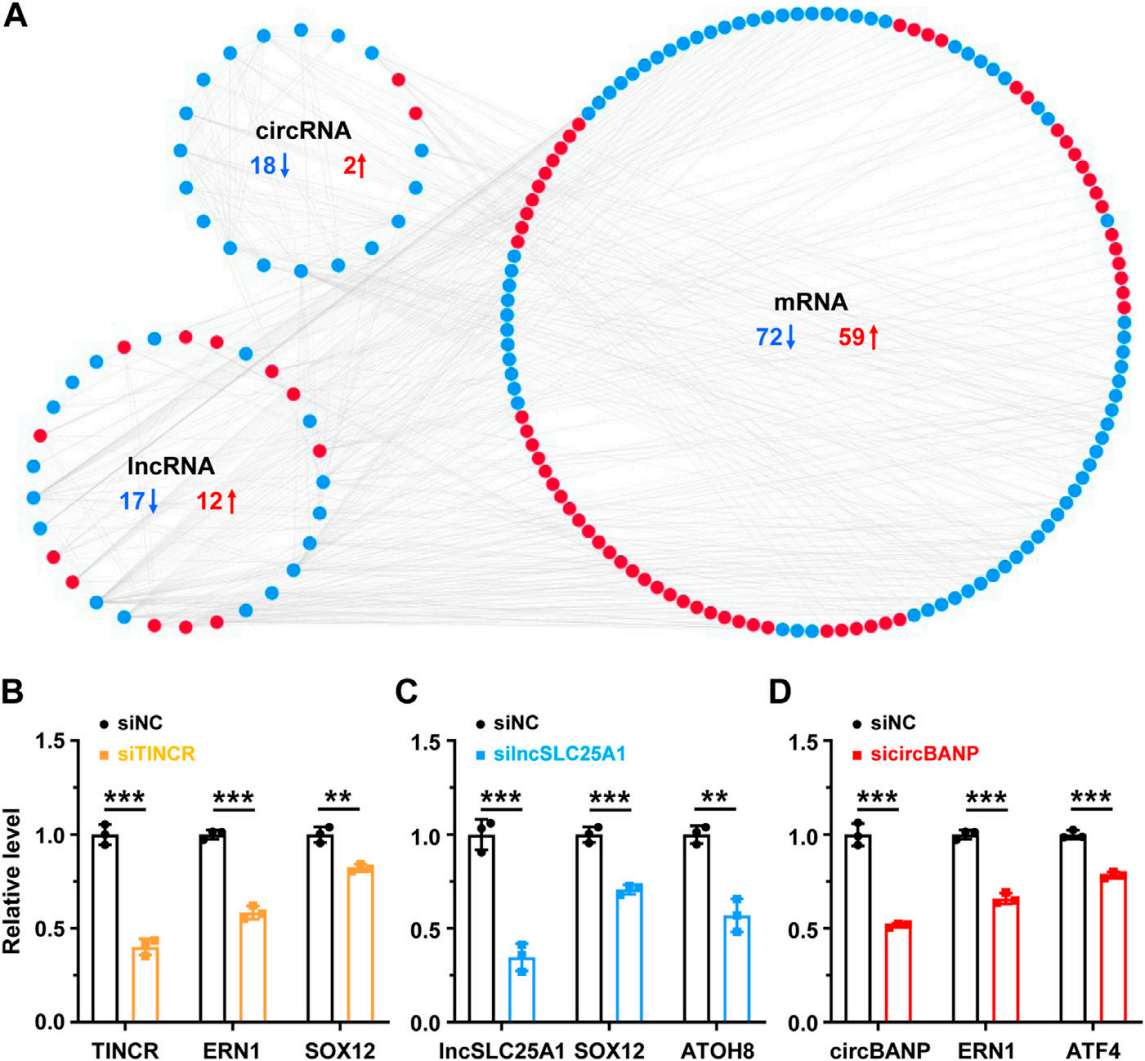
The co-expression network of lncRNA/circRNA/mRNA **(A)** The co-expression network between differentially expressed lncRNAs, circRNAs and mRNAs sensitive to both TG and GD treatments (correlation coefficient absolute value ≥ 0.95). Red, upregulated; blue, downregulated **(B)** RT-qPCR analysis of the knockdown efficiency of *TINCR* and expression levels of co-expressed *ERN1* or *SOX12* mRNA in HeLa cells treated with the siRNA against *TINCR.* siNC, siRNA with scrambled sequences; siTINCR, siRNA against *TINCR* **(C)** RT-qPCR analysis of the knockdown efficiency of *lncSLC25A1* and expression levels of co-expressed *SOX12* or *ATOH8* mRNA in HeLa cells treated with the siRNA against *lncSLC25A1.* siNC, siRNA with scrambled sequences; silncSLC25A1, siRNA against *lncSLC25A1* **(D)** RT-qPCR analysis of the knockdown efficiency of *circBANP* and expression levels of co-expressed *ERN1* or *ATF4* mRNA in HeLa cells treated with the siRNA against *circBANP.* siNC, siRNA with scrambled sequences; sicircBANP, siRNA against the junction sites of *circBANP.* Data are representative of three independent experiments and shown as mean ± SEM. **p < 0.01; ***p < 0.001 by two-tailed Student’s t-test.

The functions of lncRNAs and circRNAs are tightly related to the roles of their co-expressed protein-coding genes (Quinn and Chang, 2016; Sheng et al., 2019; Chen et al., 2022). To investigate the roles of lncRNAs and circRNAs associated with ER and metabolic stresses, we constructed a co-expression network (Spearman R ≥ 0.95 or ≤ -0.95, *p*-value < 0.01) of lncRNAs, circRNAs and their co-expressed DE mRNAs (RPM ≥10, fold change ≥2 or ≤0.5, *p*-value < 0.05) for the 51 DE lncRNAs and 39 DE circRNAs identified in both stresses according to our RNA-seq data (Figure 3A). Our analysis revealed that 29 lncRNAs (17 downregulated, 12 upregulated) and 20 circRNAs (18 downregulated, 2 upregulated) interacted with 131 DE mRNAs (72 downregulated, 59 upregulated) (Figure 3A). Notably, we found that the genomic distances of all interacted nodes in the co-expressed network were more than 100 kilobases, indicating the *trans* roles of lncRNAs and circRNAs on the co-expressed mRNAs. RNA interference (RNAi) is a widely used approach to deplete lncRNA/circRNA of interest, although there are multiple other methods (Quinn and Chang, 2016; Zhang et al., 2021). We then performed loss of function studies of three candidates (two lncRNAs and one circRNA) using RNAi to validate the co-expressed correlation between DE lncRNAs/circRNAs and mRNAs (Figures 3B–D). Small interfering RNA (siRNA)-mediated *TINCR* silencing resulted in significantly decreased expressions of its co-expressed mRNAs (*ERN1* and *SOX12*) in HeLa cells (Figure 3B). Consistently, *lncSLC25A1* knockdown decreased its co-expressed targets (*SOX12* and *ATOH8*), and *circBANP* depletion downregulated its co-expressed targets (*ERN1* and *ATF4*) (Figures 3C, D). The heatmap further demonstrated that these 131 mRNAs were significantly dysregulated after TG and GD treatments (Supplementary Figure S2A). Gene Ontology (GO) analysis revealed that these mRNAs present in the co-expressed network significantly enriched in biological processes such as response to toxic substance, response to stress, response to extracellular stimulus, regulation of RNA metabolic process, programmed cell death, PERK-mediated unfolded protein response and cellular response to glucose starvation (Supplementary Figure S2B).

### Construction of ceRNA regulatory network

**FIGURE 4:**
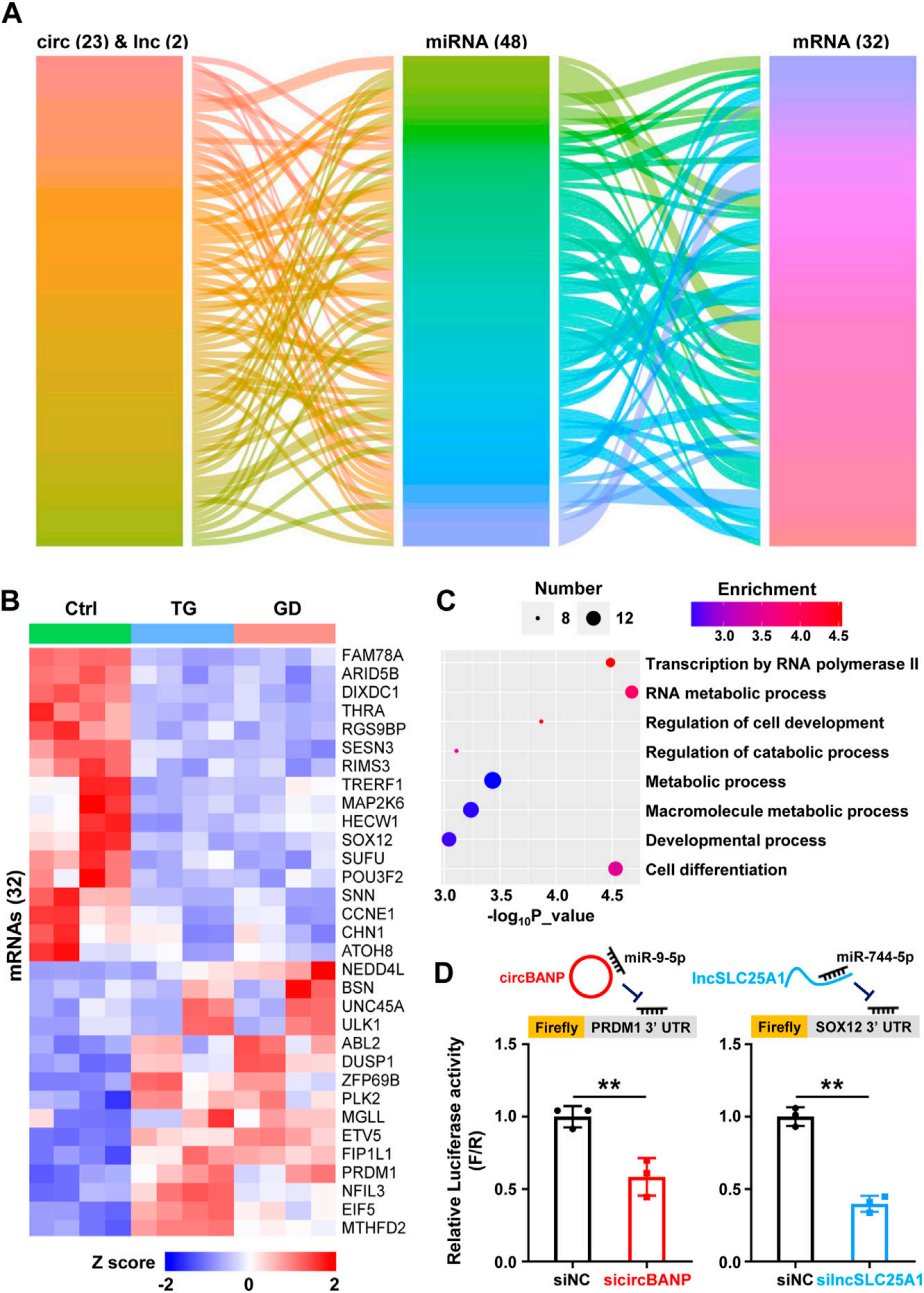
The ceRNA regulatory network **(A)** Alluvial ceRNA network was constructed based on the lncRNA/circRNA/mRNA-miRNA interactions among 51 lncRNAs and 39 circRNAs sensitive to both TG and GD treatments. All the interactions were predicted with TargetScanHuman 7.2 **(B)** The heatmap showing the levels of 32 mRNAs present in the above network in TG- or GD-treated HeLa cells. Red boxes represent upregulated mRNAs and blue boxes represent downregulated mRNAs **(C)** GO analysis revealing biological processes of 32 mRNAs present in the above network **(D)** The diagram of firefly luciferase reporters was shown (top). HeLa cells were co-transfected with sicircBANP or silncSLC25A1, the Renilla luciferase plasmid and Firefly luciferase reporter plasmids harboring the *PRDM1* or *SOX12* 3′ UTR. The ratio of Firefly (F) to Renilla (R) in relative luciferase activity was plotted. siNC, siRNA with scrambled sequences; sicircBANP, siRNA against the junction sites of *circBANP*; silncSLC25A1, siRNA against *lncSLC25A1.* Data are representative of three independent experiments and shown as mean ± SEM. **p < 0.01 by two-tailed Student’s *t*-test.

One of the molecular mechanisms for lncRNAs and circRNAs is to act as competing endogenous RNAs (ceRNAs), in which lncRNAs and circRNAs bind with miRNAs and decrease the corresponding binding of miRNAs on mRNA targets (Quinn and Chang, 2016; Chen et al., 2022). In order to explore the potential lncRNAs and circRNAs serving as ceRNAs responsive to cellular stresses *via* sequestering miRNAs and thus regulating mRNA targets, we constructed a ceRNA network among the DE lncRNAs, circRNAs, and mRNAs respond to both ER and metabolic stresses. 23 circRNAs, 2 lncRNAs, 48 miRNAs, and 32 mRNAs composed of the ceRNA regulatory network (Figure 4A). Among 32 mRNA targets, 17 were downregulated and 15 were upregulated in response to TG and GD treatments (Figure 4B). Then, we conducted GO analysis for the 32 mRNAs in the ceRNA network and found that they were associated with regulations of biological processes including transcription by RNA polymerase II, RNA metabolic process, macromolecule metabolic process, developmental process and cell differentiation (Figure 4C). To further verify the ceRNA roles of lncRNAs and circRNAs, we performed a dual-luciferase reporter assay in HeLa cells. In our network, *circBANP* interacted with *miR-9-5p* to derepress the *PRDM1* level (Figure 4D). Knockdown of *circBANP* mediated by siRNA significantly reduced the luciferase activity of Firefly with the *PRDM1* 3’ UTR region containing the *miR-9-5p* binding site (Figure 4D). The *lncSLC25A1*-*miR-744-5p*-*SOX12* axis exhibited a similar result according to our experiments (Figure 4D). Although, we have to point out that these were just results from overexpression and RNAi experiments, and further validations are required to examine the ceRNA regulatory network.

### RNA-RBP interaction network

**FIGURE 5:**
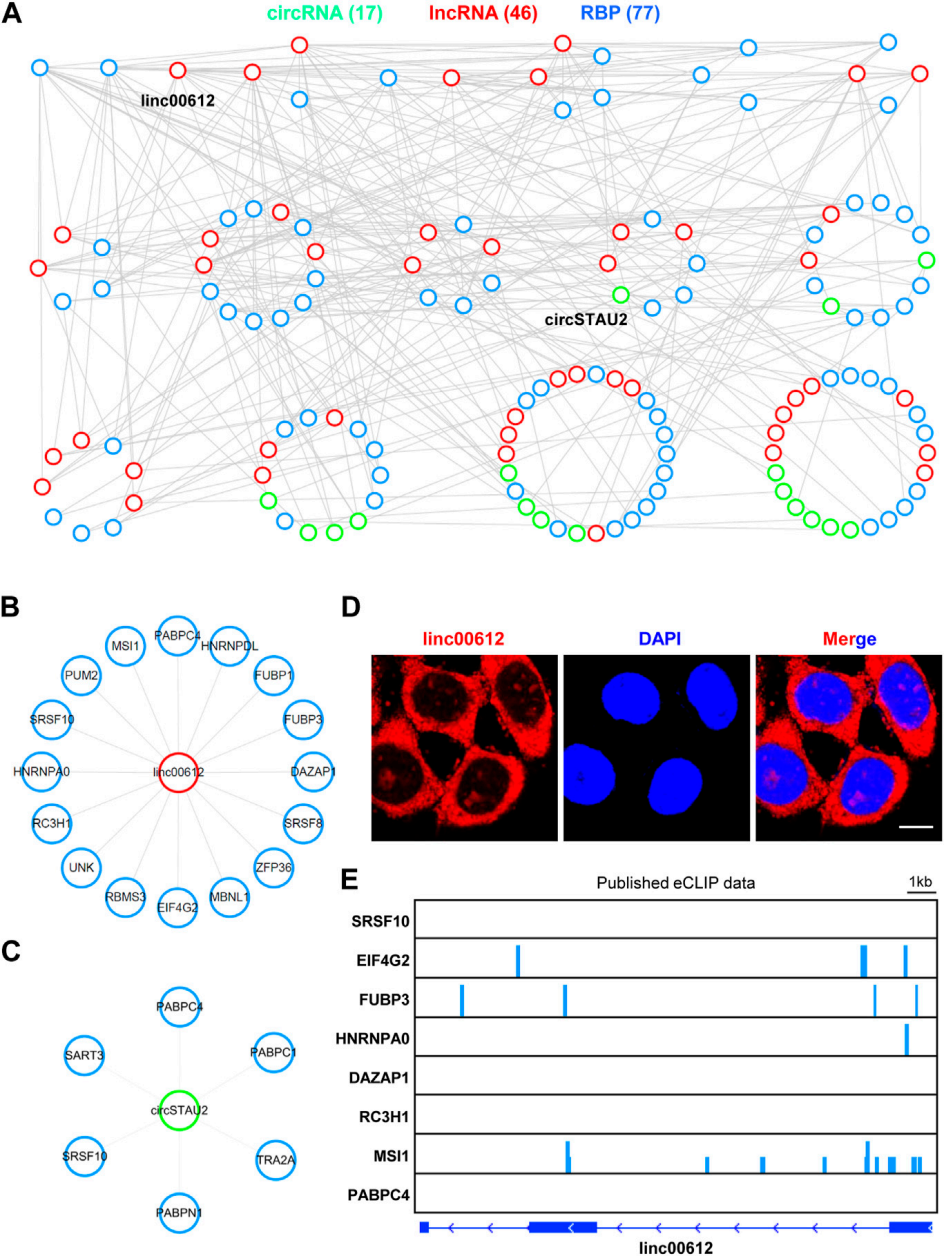
The RNA-RBP interactome map **(A)** The lncRNA/circRNA-RBP network was constructed according to the interactions among 51 lncRNAs and 39 circRNAs sensitive to both TG and GD treatments. All the interactions were predicted with RBPmap. The network was plotted by the ranked degrees. RBP, RNA binding protein **(B)** *Linc00612* interacted with 16 RBPs in the above network **(C)** *CircSTAU2* interacted with 6 RBPs in the above network **(D)** RNA FISH with the antisense probe showed the subcellular localization of *linc00612* (red) in HeLa cells. Nuclei (blue) were stained with DAPI. Scale bar, 10 μm **(E)** The analyses of eight published eCLIP-seq data indicating the binding signals on *linc00612.*

Given that lncRNAs and circRNAs could interact with RBPs to exert their crucial functions (Chen et al., 2022; Wang et al., 2022), we predicted the potential binding RBPs of both DE lncRNAs and DE circRNAs in ER or metabolic stresses *via* RBPmap, a tool mapping the interacting RBPs for RNAs of interest (Paz et al., 2014), and constructed the lncRNA/circRNA-RBP network based on the predicted interactions (Figure 5A). The network consisted of 17 circRNAs, 46 lncRNAs and 77 RBPs (Figure 5A). We also noticed that among the network, *linc00612* and *circSTAU2* interacted with the most RBPs among lncRNAs and circRNAs, respectively (Figure 5A). *Linc00612* interacted with 16 different RBPs such as SRSF8, HNRNPDL, PUM2, and *circSTAU2* interacted with 6 distinct RBPs such as PABPC1, SART3, SRSF10 (Figures 5B, C). Furthermore, the mRNA levels of 77 RBPs were dynamic after TG and GD treatments (Supplementary Figure S3A). GO analysis for these 77 RBPs indicated that they were related to biological processes including RNA 3’ end processing, mRNA stability involved in response to stress, regulation of translation, gene silencing by miRNA, nuclear export, mRNA splicing *via* spliceosome, etc. (Supplementary Figure S3B). RNA fluorescence *in situ* hybridization (FISH) displayed that *linc00612* mostly localized to the cytoplasm, and a small portion resided in the nucleus in HeLa cells (Figure 5D), implying that *linc00612* might possess both cytoplasmic and nuclear roles. In addition, integrative analysis of published eCLIP-seq data of RBPs revealed that EIF4G2, FUBP3, HNRNPA0, and MSI1 demonstrated binding signals on *linc00612* (Figure 5E).

## Discussion

Prolonged exposures to stresses are tightly associated with numerous diseases including cancers and metabolic disorders (Urra et al., 2016; Galluzzi et al., 2018); thus, clarifying cellular responses and common responsive factors underneath multiple stresses is of great importance. Systematic investigations about stress response networks are still required (Galluzzi et al., 2018; Chen et al., 2021). Here, we have applied RNA-seq to identify the DE lncRNAs and circRNAs in two cellular stresses and tentatively revealed the regulatory interactions and potential functional mechanisms of these RNAs, shedding new insights into cellular responses and providing some indications for disease relevancies.

RNA-seq followed by bioinformatics analyses is a powerful tool to characterize aberrantly expressed protein-coding and non-coding genes at the transcriptome level (Mortazavi et al., 2008). The studies about stress-related RNAs are accumulating, and these transcripts may be correlated with stress or anti-stress roles (Settembre et al., 2011; Wiseman et al., 2022). Increasing evidence has demonstrated that lncRNAs and circRNAs along with mRNAs are extensively involved in cellular responses, with some of them being identified as stress sensors (Li et al., 2019; De Troyer et al., 2020; Hetz et al., 2020; Zhu et al., 2022). However, systematic identification of differentially expressed RNAs, especially circRNAs, under two distinct cellular stimuli, is limited.

In the present study, we have performed transcriptomic profiles of HeLa cells treated with TG or GD and identified 51 lncRNAs and 39 circRNAs sensitive to both stresses (Figure 1). We have validated 12 DE RNA candidates, which are also in high accordance with the RNA-seq (Figure 2). Several of them are known to have substantial involvements in physiology and diseases (Hong et al., 2020; Kretz et al., 2013; Marín-Béjar et al., 2017; Simchovitz et al., 2020; Zheng et al., 2020). For example, *lincPINT*, which is upregulated during TG or GD treatment, plays crucial roles in many diseases, such as neurodegeneration and cancers (Marín-Béjar et al., 2017; Simchovitz et al., 2020). *TINCR* is decreased in two stress conditions and has been reported to contribute to various cellular processes, including cell proliferation, apoptosis, autophagy, invasion and metastasis (Kretz et al., 2013; Zheng et al., 2020). *CircCRIM1* has been reported to promote nasopharyngeal carcinoma (NPC) metastasis and docetaxel chemoresistance *via* serving as a ceRNA against *miR-422a* to improve the *FOXQ1* level (Hong et al., 2020). All these results imply that these DE lncRNAs and circRNAs identified in both stresses might behave as pivotal regulators responsive to cellular stresses. To explore the regulatory functions of lncRNAs and circRNAs, we have constructed the co-expressed network, the ceRNA network, and the RNA-RBP interaction map (Figures 3–5). Furthermore, GO analysis has revealed the potential roles of lncRNAs and circRNAs present in these networks in the corresponding biological processes. Hopefully, this study can provide helpful aspects for future investigations of stress responses.

NcRNA-mRNA interaction is one of the most frequently studied molecular mechanisms of ncRNAs (Quinn and Chang, 2016; Chen et al., 2022). We have constructed a co-expressed network for 51 lncRNAs and 39 circRNAs sensitive to ER and metabolic stresses (Figure 3A). GO analysis has revealed mRNAs in the network are enriched on biological pathways such as response to toxic substance, response to stress, response to extracellular stimulus, programmed cell death, PERK-mediated unfolded protein response and cellular response to glucose starvation (Supplementary Figure S2B). These GOs strongly indicate the engagement of lncRNAs and circRNAs in stress responsive processes, and also to some degree provide proof for the constructed network.

We have also constructed a ceRNA network based on the lncRNA/circRNA-miRNA-mRNA interaction and performed experimental data to verify the ceRNA roles of lncRNAs and circRNAs (Figure 4). According to the result of GO analysis, 23 circRNAs, 2 lncRNA and 48 miRNAs composed of the network, which may participate in the processes including transcription by RNA polymerase II, RNA metabolic process and cell differentiation (Figure 4C). From a previous publication, *miR-423-5p* in the network is indeed upregulated under ER stress, and exerts its function by targeting *CDKN1A* (Dai et al., 2015). Although accumulating evidence is supportive of the ceRNA mechanism, concerns about this concept have been raised (Bosson et al., 2014; Denzler et al., 2014; Tay et al., 2014; Chen et al., 2022). The molecular ratio and the endogenous expression levels need to be carefully evaluated, and more examples with convincing physiological data should be provided to further prove ceRNA regulations.

RNA-protein interaction is another functional mechanism of RNAs (Licatalosi and Darnell, 2010; Chen et al., 2022). Increasing evidence has demonstrated that lncRNAs and circRNAs can exert their functions through modulating or sequestering one or more RBPs (Lee et al., 2016; Wang et al., 2021). Based on the RNA-protein interactions, we have constructed the RNA-RBP interactome map (Figure 5), and this type of interactome map is still scarce. In the network, *linc00612* and *circSTAU2* interact with the maximum number of proteins among lncRNAs and circRNAs, respectively (Figures 5A–C). *Linc00612* has been reported to promote the progressions of osteosarcoma and bladder cancer (Miao et al., 2019; Zhou et al., 2020). *Linc00612* binds to 16 RBPs with distinct functional roles, such as transcription (HNRNPDL, FUBP1), splicing (SRSF8, SRSF10, MBNL1), RNA stability (ZFP36, RC3H1, PABPC4), and translation (EIF4G2, UNK, MSI1). Cellular localization of an ncRNA is critical for its functionality. *Linc00612* mostly localizes to the cytoplasm, which may be associated with its role in regulation of translation. Meanwhile, a small portion of *linc00612* resides in the nucleus, which may be responsible for its regulatory roles in transcription and splicing. *CircSTAU2* interacts with splicing-related proteins (TRA2A, SRSF10, SART3) and RNA stability-related proteins (PABPC1, PABPC4, PABPN1). *Linc00612* and *circSTAU2* both interact with SRSF10 and PABPC4, implying that both proteins may be key factors in response to cellular stresses.

We would also like to point out several limitations of this study. We cannot rule out that the DE lncRNAs/circRNAs identified under the cellular stresses tested have cell-type specificity. Although we have found a series of DE lncRNAs and circRNAs in cellular stress responses, investigations about functions of individual ncRNA of interest, and the molecular mechanism are required. Moreover, the lncRNA/circRNA regulatory networks need further validation with more experimental explorations.

## Conclusion

In summary, our study has identified a number of DE lncRNAs and circRNAs responsive to ER and metabolic stresses, and has constructed associated regulatory networks to provide novel insights for functional and mechanistic explorations of ncRNAs under cellular stresses.

## Data Availability

The datasets presented in this study can be found in online repositories. The names of the repository/repositories and accession number(s) can be found in the article/Supplementary Material.
