# Supplementary material for: Identification of long non-coding RNA and circular RNA associated networks in cellular stress responses
Source: Front Genet. 2023 Feb 10;14:1097571. doi: 10.3389/fgene.2023.1097571 (PMC9953141; doi:10.3389/fgene.2023.1097571)
Supplement: Supplementary file 2 [file DataSheet1.PDF]

Figure S1. Transcriptome analyses of HeLa cells treated with TG and GD, related to Figure 1.

(A) RT-PCR gels of *XBP1* and *FST* mRNAs in HeLa cells treated with TG and GD, respectively. *XBP1s* and *FST* mRNAs used as the positive controls are known to be upregulated upon TG and GD treatments, respectively. The *ACTB* mRNA is used as the loading control. TG, thapsigargin; GD, glucose deprivation; Ctrl, control; *XBP1s*, spliced *XBP1*; *XBP1u*, unspliced *XBP1*.

(B) Schematic workflow for RNA-seq and bioinformatic analyses. CIRI2 and find_circ were applied to identify high-confidence BSJs for circRNAs. BSJ, back-splicing junction.

(C) PCA plots of the RNA-seq dataset demonstrating the similarities/differences among TG, GD and Ctrl groups with four replicates. Percentages represented variance captured by each principal component 1 and 2 in each analysis. The BSJ reads of circRNAs and linear RNA reads were counted for PCA analysis. PCA, principal component analysis.

(D) General description of RNA-seq data.

Figure S2. The co-expression network of lncRNA/circRNA/mRNA, related to Figure 3.

(A) The heatmap showing the expression levels of 131 mRNAs present in the lncRNA/circRNA-mRNA co-expression network in TG- or GD-treated HeLa cells. Red boxes represent upregulated mRNAs and blue boxes represent downregulated mRNAs.

(B) GO analysis revealing biological processes of 131 mRNAs present in the above network.

Figure S3. The RNA-RBP interactome map, related to Figure 5.

(A) The heatmap demonstrating the mRNA levels of 77 RBPs present in the RNA-RBP interactome network in TG- or GD-treated HeLa cells. Red boxes represent upregulated mRNAs and blue boxes represent downregulated mRNAs.

(B) GO analysis revealing biological processes of 77 mRNAs present in the above network.

Fig. S1

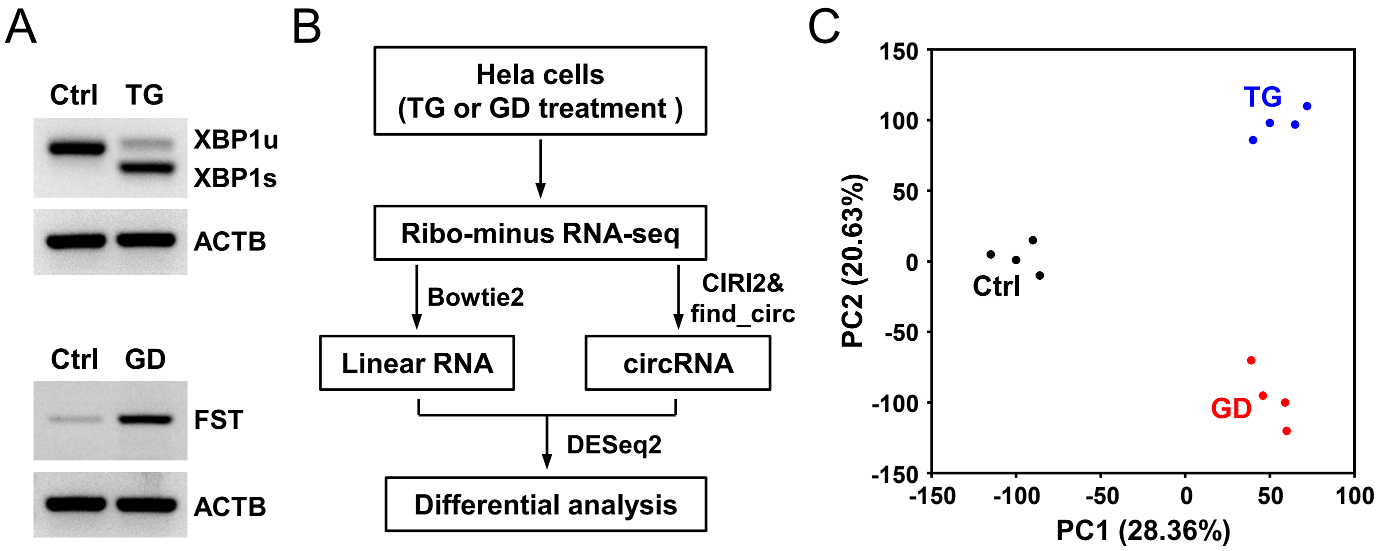

D

Sample	Ctrl1	Ctrl2	Ctrl3	Ctrl4	TG1	TG2	TG3	TG4	GD1	GD2	GD3	GD4
Data size (G)	16.09	14.82	12.51	9.46	14.81	14.93	7.89	13.14	15.29	15.63	5.03	5.59
Depth	3.6X	3.3X	2.8X	2.1X	3.2X	3.2X	1.8X	2.9X	3.3X	3.4X	1.1X	1.2X
BSJ reads (‰)	1.15	1.22	0.98	0.99	1.19	1.16	0.82	0.81	1.41	1.19	0.84	0.87

Fig. S2

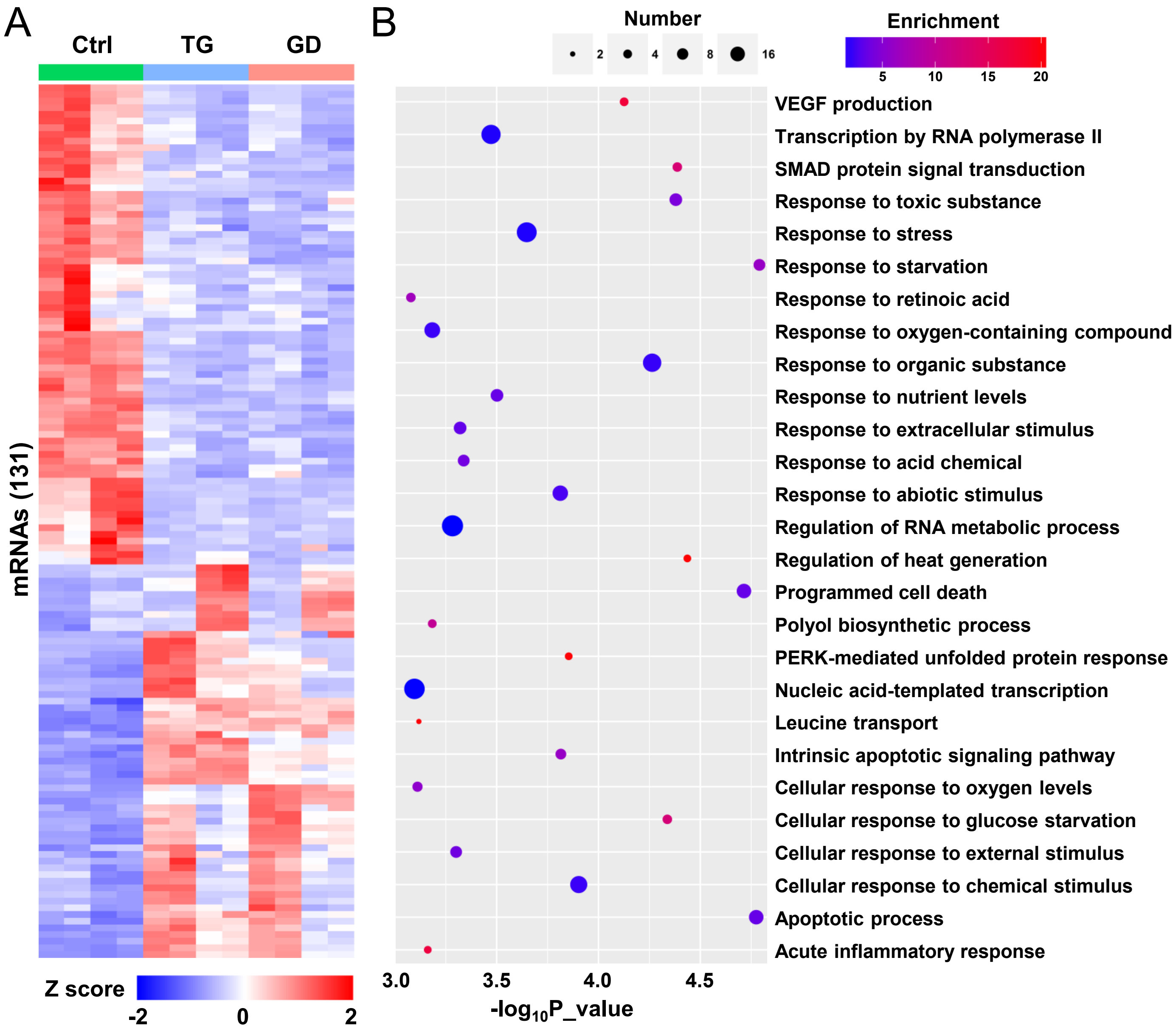

A
Ctrl
TG
GD
mRNAs (131)
Z score
-2
0
2
B
Number
2
4
8
16
Enrichment
5
10
15
20
VEGF production
Transcription by RNA polymerase II
SMAD protein signal transduction
Response to toxic substance
Response to stress
Response to starvation
Response to retinoic acid
Response to oxygen-containing compound
Response to organic substance
Response to nutrient levels
Response to extracellular stimulus
Response to acid chemical
Response to abiotic stimulus
Regulation of RNA metabolic process
Regulation of heat generation
Programmed cell death
Polyol biosynthetic process
PERK-mediated unfolded protein response
Nucleic acid-templated transcription
Leucine transport
Intrinsic apoptotic signaling pathway
Cellular response to oxygen levels
Cellular response to glucose starvation
Cellular response to external stimulus
Cellular response to chemical stimulus
Apoptotic process
Acute inflammatory response
3.0
3.5
4.0
4.5
-log10P_value

Fig. S3

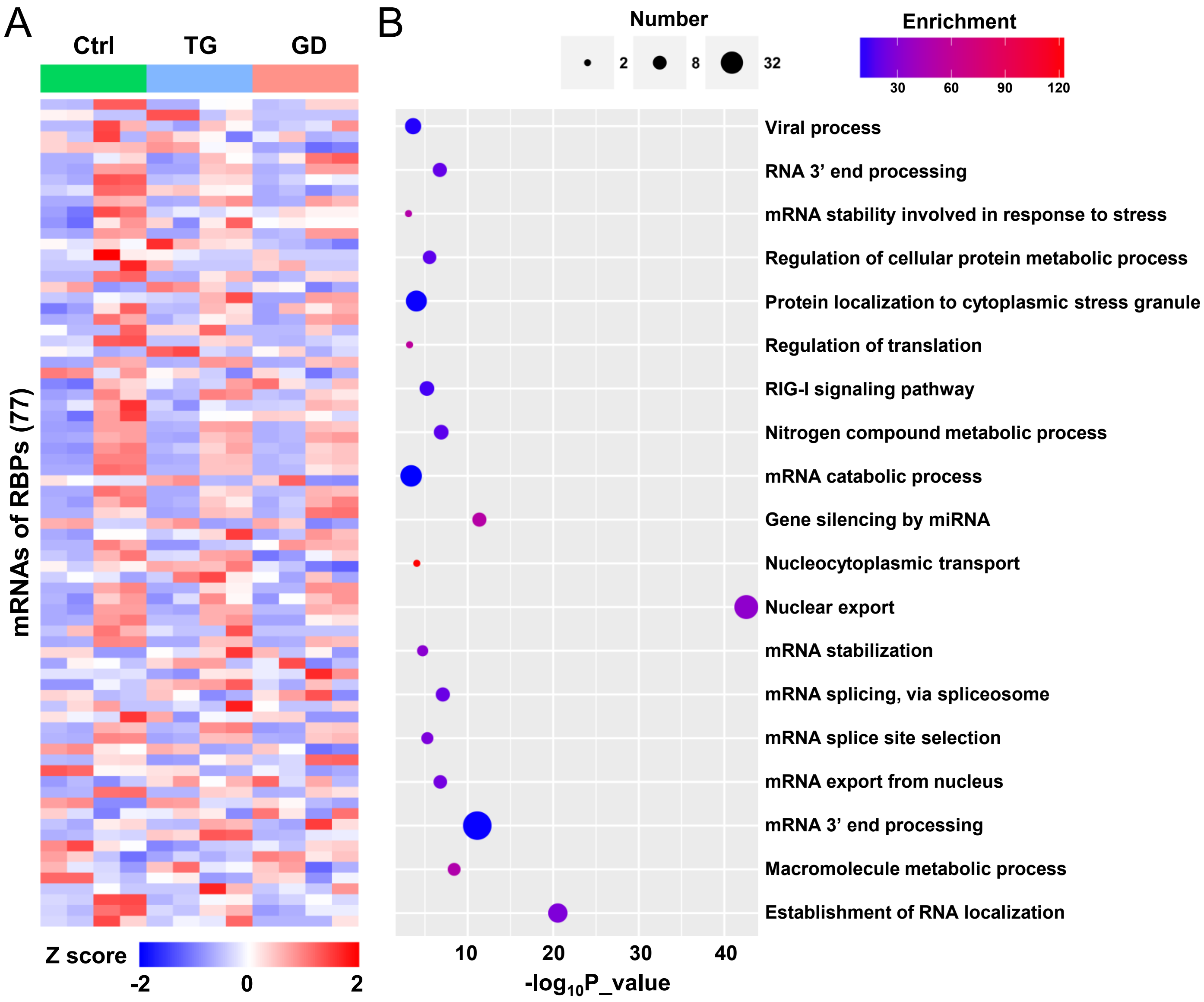
A
Ctrl
TG
GD
mRNAs of RBPs (77)
Z score
-2
0
2
B
Number
2
8
32
Enrichment
30
60
90
120
Viral process
RNA 3' end processing
mRNA stability involved in response to stress
Regulation of cellular protein metabolic process
Protein localization to cytoplasmic stress granule
Regulation of translation
RIG-I signaling pathway
Nitrogen compound metabolic process
mRNA catabolic process
Gene silencing by miRNA
Nucleocytoplasmic transport
Nuclear export
mRNA stabilization
mRNA splicing, via spliceosome
mRNA splice site selection
mRNA export from nucleus
mRNA 3' end processing
Macromolecule metabolic process
Establishment of RNA localization
10
20
30
40
-log10P_value
