# Supplementary material for: Identification of long non-coding RNA and circular RNA associated networks in cellular stress responses
Source: Front Genet. 2023 Feb 10;14:1097571. doi: 10.3389/fgene.2023.1097571 (PMC9953141; doi:10.3389/fgene.2023.1097571)
Supplement: Supplementary file 3 [file Table3.DOCX]

**Table 3. Oligonucleotide sequences used in this study.**

| Gene ID | Oligonucleotide sequence (5' -3') | Product size (bp) |
| --- | --- | --- |
| lincPINT | F: CTATTTGGTGCTACTTCTAC  R: CAGGCATTCAGCCAGCACTC | 171 |
| lncSARS1 | F: GTAGATAGGTGGTGAGTTCTT  R: AAGACAGAAAATATTCACGC | 170 |
| lncZFAS1 | F: TACAACCTTCGATCTCCTGA  R: CAGTTTCTGTTACAAGGCAG | 177 |
| BASP1-AS1 | F: TCGCTCAGACTTAGGGAGCT  R: CAACTCGTTTGCAGCGGCGC | 184 |
| lncNT5C3B | F: GTCAGTGGTTCTCTCGGGTC  R: CAAACCTGCTCAAGGTCATG | 165 |
| TINCR | F: CTAGATCTCACTCCAGGGTC  R: CCGATAGGCTGGGCAGGAGC | 146 |
| lncSLC25A1 | F: GACTGCGTGCGGCAGACGGT  R: TCCCGCACAATCTCCCTAAC | 178 |
| circCRIM1 | F: CATAGTCTCTCGTGGCGATGG  R: ATAAGGTTTTCATTGCATGG | 120 |
| circHBS1L | F: GAGTGCAGAGTTTGAAGGAC  R: GTTTGAAACAGAATTGGAAG | 161 |
| circPTBP2 | F: ATTGGACCCAGCTATTGCTG  R: CGAATATGAAGTACACGAG | 126 |
| circABL2 | F: GAAGGAAGATACCATGGAGG  R: TGATGGTGTACAAGCTCTGC | 145 |
| circBANP | F: AAGTCCAGATCACGCAGGAC  R: GGCAGTTGGTGCTGATGTGC | 170 |
| circCEP72 | F: CGTGAGAGCAAGCGAGCGG  R: CAATGAGCCAAGATTGCGTC | 156 |
| ACTB | F: CCAACACAGTGCTGTCTGG  R: GAGTACTTGCGCTCAGGAG | 130 |
| XBP1 | F: GTTGAGAACCAGGAGTTAAG  R: GACTCTGAATCTGAAGAGTC | 220 (XBP1u)  194 (XBP1s) |
| FST | F: CTGGGAACTGCTGGCTCCGT  R: TTTACAGGGGATGCAGTTGG | 191 |
| ERN1 | F: ATGTGGAAGAGCCTGCCTTTC  R: GCTTTTTACCCATGTAGAGG | 165 |
| SOX12 | F: ACATGCACAACGCCGAGATC  R: TACTTGTAGTCCGGGTAATC | 136 |
| ATOH8 | F: CAATAACCACCAGGATTCCT  R: AGCACGGCACCTGCTTCCTG | 179 |
| ATF4 | F: ACAGCAAGGAGGATGCCTTC  R: AGAGATCACAAGTGTCATCC | 150 |
| PRDM1 3' UTR | F: GATCGCCGTGTAATTCTAGA  GAAATGATGTCTTATCTAATG  R: TCTGCTCGAAGCGGCCGGCC  CAATTTGCACATAAATAACA | 1096 |
| SOX12 3' UTR | F: GATCGCCGTGTAATTCTAGAC  CATCTCCCGCGCAGCCTGC  R: TCTGCTCGAAGCGGCCGGCC  TACAGGTAGTGAGGGCAAG | 1388 |
| linc00612 FISH probe | F: GACATCTGTGCCTCGCGTC  R: TGCCCGACTACACTGCTTC | 295 |
| siNC | S: UUCUCCGAACGUGUCACGUTT  AS: ACGUGACACGUUCGGAGAATT | -- |
| siTINCR | S: GCUGCUGCUCGUCAGCGACTT  AS: GUCGCUGACGAGCAGCAGCTT | -- |
| silncSLC25A1 | S: CACCGAGUACGUGAAGACGTT  AS: CGUCUUCACGUACUCGGUGTT | -- |
| sicircBANP | S: GGACGGUCAGAGGACUACCTT  AS: GGUAGUCCUCUGACCGUCCTT | -- |
